# Recurrent dislocation of the patella accompanying hypotrochlea of the femur and malalignment of the patella

**DOI:** 10.3109/03009734.2011.596291

**Published:** 2011-10-29

**Authors:** Akira Horikawa, Hiroyuki Kodama, Naohisa Miyakoshi, Shin Yamada, Seiya Miyamoto

**Affiliations:** ^1^South Akita Orthopedic Clinic, Seiwakai, 96-2 Kaidousita, Syowa-Ookubo, Katagami 018-1401, Japan; ^2^Department of Orthopedic Surgery, Akita University Graduate School of Medicine, 1-1-1 Hondo, Akita 010-8543, Japan; ^3^Department of Orthopedic Surgery, Nakadori General Hospital, 3-15 Minamidoori-Misono, Akita 010-8577, Japan

**Keywords:** Dislocations, knee, patella

## Abstract

This case report describes a rare case of recurrent dislocation of the patella which was accompanied with trochlear dysplasia and malalignment of the patella in a 15-year-old girl. She complained of hemoarthrosis and recurrent patellar dislocation in the early knee flexion phase. Plain radiography and computed tomography (CT) showed patellar malalignment (quadriceps angle 20°) and severe dysplasia of the trochlea of the femur (sulcus angle 170°). Surgery was performed, consisting of trochleoplasty in addition to proximal and distal realignment. Trochleoplasty was undertaken using a modified Dejour technique. After surgery, the patient complained of joint contracture. Arthroscopic release of fibrous tissue relieved symptoms and obtained normal range of motion without patellar dislocation. Postoperative radiography and CT demonstrated improvement of the quadriceps angle (10°) and sulcus angle (140°).

## Introduction

Although biomechanics and surgical treatments have been examined for recurrent dislocation of the patella, few studies have reported on the combination of trochlear dysplasia and recurrent patellar instability. In addition, treatment methods have only been reported in a few cases. We present herein a case with successful surgical outcomes for the treatment of recurrent dislocation of the patella with trochlear dysplasia and malalignment of the patella.

## Case report

**Figure 1.**
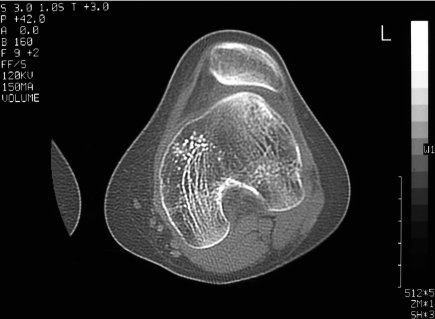
Preoperative computed tomography demonstrating marked trochleodysplasia of the femur, with a sulcus angle of 170°.

A 15-year-old girl who had complained of recurrent patellar dislocation for more than 5 years presented with left knee hemoarthrosis. Physical examination revealed patellar dislocation at slight knee flexion of approximately 30° that was easily reduced without pain. No signs of apprehension, joint laxities, or systematic disease were observed. Plain radiography showed a lateralized tibial tubercle with increased quadriceps angle (Q angle) of 20° and dysplasia of the trochlea as defined by the ‘crossing sign’ (1) without patella alta. Computed tomography (CT) also demonstrated marked trochleodysplasia of the femur, with a sulcus angle of 170° (Figure 1).

**Figure 2.**
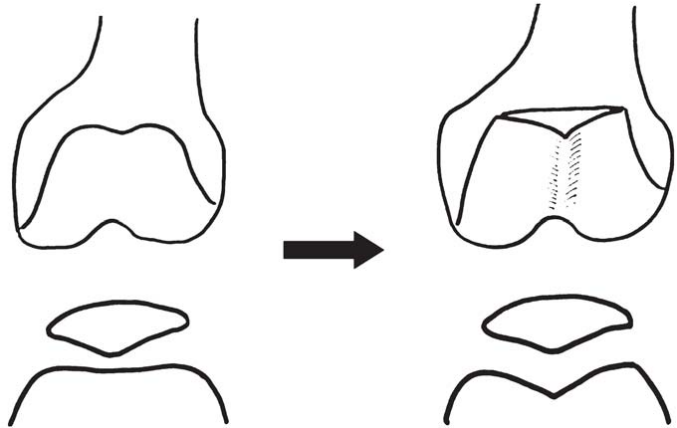
Schema of the modified Dejour technique. After shaving the subchondral bone, an incision from the aspect of the groove was made. A subchondral trench was then cut and the flap undermined, followed by impaction using finger pressure and screw fixation.

Arthroscopic examination showed a flat, hypoplastic trochlea of the femur and frequent patellar dislocation with knee motion. Surgery consisted of trochleoplasty, followed by proximal and distal realignment. Trochleoplasty was performed as a modified Dejour technique (2). This technique involved: 1) shaving of the subchondral bone; 2) making an incision from the aspect of the groove to the anterior femoral cortex; 3) cutting a subchondral trench; 4) undermining the flap to allow collapse of the osteochondral flap; 5) impaction using finger pressure; and 6) fixation of the cartilage by screw as advocated by Donell et al. (2) (Figure 2). Proximal realignment involved medical retinaculum suture (reefing), as described by Insall et al. (3). Distal realignment comprised medialization of the tibial tuberosity according to Fulkerson's osteotomy (4), transferring the tuberosity site 15 mm medially.

Following surgery, a long leg plaster cast was applied for 2 weeks. Physical therapy including strengthening quadriceps muscle and improvement of range of motion was started after cast removal, by referring to Kobayashi and Ou's rehabilitation protocol (5). Partial weight-bearing was allowed from 4 weeks postoperatively. Although no malalignment of patella was seen on plain radiography, the patient complained of joint contracture from 0° extension to 40° flexion. Considering these symptoms, second-look arthroscopy was performed.

**Figure 3.**
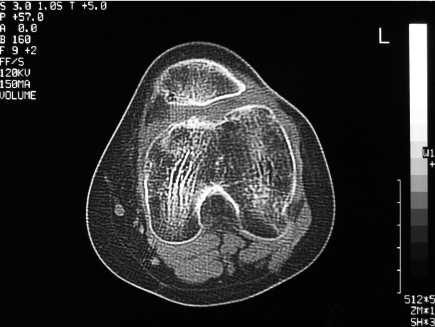
Postoperative computed tomography demonstrating no malalignment of the patella and improvement of sulcus angle from 170° to 140°.

A second-look arthroscopic examination demonstrated a good-shaped trochlear groove without cartilage defect. However, fibrous adhesion was seen mainly in the suprapatellar pouch. After arthroscopic release of the lateral retinaculum and patellar tendon, knee motion normalized. Physical examination more than 2 years after surgery showed a 0–150° range of motion of the knee without pain. The improved Q angle was 10°, and the patient did not complain of patellar dislocation. Postoperative CT demonstrated no malalignment of the patella, and sulcus angle had improved from 170° to 140° (Figure 3).

## Discussion

The treatment of trochlear dysplasia with malalignment of the patella needs to achieve realignment of the patella, such as transfer of the tibial tuberosity in patients with open epiphysis, which might lead to remodeling of the trochlear groove (6). Conversely, trochleoplasty should be considered carefully in cases with closed epiphysis in which remodeling following the realignment procedure may not be expected (7–10). Regarding these procedures, Kobayashi and Ou indicated trochleoplasty for cases with sulcus angle >150° with closed epiphysis and patella dislocation or subluxation in the early flexion phase (5). Considering these treatment options, trochleoplasty was indicated for the present case with a closed epiphysis.

Although trochleoplasty is a good surgical procedure for the treatment of trochlear dysplasia, some problems after this surgery have been reported. Kuroda et al. suggested that raising the lateral surface of the trochlea will further increase joint pressure, and thereby increase the risk of arthritis developing in the case of trochleoplasty (11). To avoid this eventuality, we chose the Dejour technique (8), which was unaccompanied by elevation of the lateral surface of the trochlea. Furthermore, we performed both proximal and distal realignment to expect to distribute joint pressure equally between the medial and lateral patellofemoral joint. Koeter et al. (12) and Schottle et al. (10) supported proximal realignment such as medial reefing and lateral release, while Donell et al. (2) and Fulkerson (4) supported distal realignment such as tibial transfer when performing trochleoplasty.

Another reason why we performed both proximal and distal realignment procedure was because the patient showed severe malalignment of the patella (Q angle 20°). For proximal realignment, we adopted the medial reefing procedure proposed by Insall et al. (3). We did not add the lateral release because the lack of balance between medial and lateral patellofemoral joint pressure might occur after additional lateral release (13). However, our case showed joint contracture similar to arthrofibrosis following immobilization in the plaster cast, in the same way as described by Donell et al. (2). Ochi (13) suggested that medial reefing and advancement of vastus medialis increased the patellofemoral joint pressure. For distal realignment, Utting et al. (14) recommended that medialization of the tibial tubercle should be carried out when the offset of the tuberosity from the trochlear groove exceeds 18 mm, and we followed that methodology. They also suggested reconstruction of the medial patellofemoral ligament or medial soft tissue reefing for persistent subluxation of the patella despite a normal tibial tuberosity (14). However, some cases with knee joint stiffness were treated using medial reefing. We used medial reefing although the offset of the tuberosity from the trochlear groove had exceeded 18 mm. This may also have led to adhesion of the patellar tendon to surrounding tissues and subsequent joint contracture. In addition, Verdonk et al. (1) pointed out that joint arthrofibrosis might be caused by the procedure of distal realignment in cases treated using trochleoplasty. Consequently, in the present case, combination of proximal realignment (medial reefing without lateral release) and distal realignment (tibial transfer) might have increased patellofemoral joint pressure compared to either alone, and it might have led to arthrofibrosis during plaster immobilization.

Trochleoplasty primarily affects the congruency of the patellofemoral joint. However, additional proximal and/or distal realignment should also be considered. Fulkerson (4) and Arendt et al. (15) emphasized that correction of dysplastic factors and realignment should be considered when minimally invasive surgery fails. According to our successful experience in the present case, although postoperative fibrous adhesions should be avoided, one-stage surgery combining trochleoplasty, proximal realignment, and distal realignment can be used as an effective treatment option for patients with recurrent patellar dislocation due to severe patellar malalignment and trochlear dysplasia.

In summary, we have described a rare case with recurrent dislocation of the patella resulting from trochlear dysplasia of the femur and malalignment of the patella. This patient underwent surgical treatments including trochleoplasty and proximal and distal realignments. Postoperative fibrous contracture occurred, but normal range of motion was obtained after arthroscopic release. Physical examination 2 years after surgery showed excellent clinical and radiological results, and the patient had no complaints of patellar dislocation.
